# A Systematic Review and Meta-Analysis of the Efficacy and Safety of Intermittent Preventive Treatment of Malaria in Children (IPTc)

**DOI:** 10.1371/journal.pone.0016976

**Published:** 2011-02-14

**Authors:** Anne L. Wilson, on behalf of the IPTc Taskforce

**Affiliations:** London School of Hygiene and Tropical Medicine, London, United Kingdom; The George Washington University Medical Center, United States of America

## Abstract

**Background:**

Intermittent preventive treatment of malaria in children less than five years of age (IPTc) has been investigated as a measure to control the burden of malaria in the Sahel and sub-Sahelian areas of Africa where malaria transmission is markedly seasonal.

**Methods and Findings:**

IPTc studies were identified using a systematic literature search. Meta-analysis was used to assess the protective efficacy of IPTc against clinical episodes of falciparum malaria. The impact of IPTc on all-cause mortality, hospital admissions, severe malaria and the prevalence of parasitaemia and anaemia was investigated. Three aspects of safety were also assessed: adverse reactions to study drugs, development of drug resistance and loss of immunity to malaria. Twelve IPTc studies were identified: seven controlled and five non-controlled trials. Controlled studies demonstrated protective efficacies against clinical malaria of between 31% and 93% and meta-analysis gave an overall protective efficacy of monthly administered IPTc of 82% (95%CI 75%–87%) during the malaria transmission season. Pooling results from twelve studies demonstrated a protective effect of IPTc against all-cause mortality of 57% (95%CI 24%–76%) during the malaria transmission season. No serious adverse events attributable to the drugs used for IPTc were observed in any of the studies. Data from three studies that followed children during the malaria transmission season in the year following IPTc administration showed evidence of a slight increase in the incidence of clinical malaria compared to children who had not received IPTc.

**Conclusions:**

IPTc is a safe method of malaria control that has the potential to avert a significant proportion of clinical malaria episodes in areas with markedly seasonal malaria transmission and also appears to have a substantial protective effect against all-cause mortality. These findings indicate that IPTc is a potentially valuable tool that can contribute to the control of malaria in areas with markedly seasonal transmission.

## Introduction

Intermittent preventive treatment of malaria (IPT) refers to the administration of a full therapeutic course of an anti-malarial drug to the whole of a population at risk, whether or not they are known to be infected, at specific times, with the aim of preventing mortality or morbidity from malaria [1]. IPT with sulphadoxine-pyrimethamine (SP) is recommended by the WHO for use in pregnant women (IPTp) and has recently been recommended by the WHO for use in infants (IPTi), delivered alongside vaccines within the context of the routine Expanded Programme on Immunisation [2]. The decision by WHO to recommend IPTi in areas where there is a significant malaria burden in infants and where parasites are still sensitive to SP was made on the basis of data including a pooled analysis of six IPTi studies which demonstrated a 30% protective efficacy (PE) against episodes of clinical malaria [3]. In areas of markedly seasonal malaria transmission, such as the Sahel and sub-Sahel regions of Africa, the main burden of malaria is in older children rather than infants, and the risk of clinical malaria is restricted largely to a few months each year [4], [5]. In such areas, administration of IPT to children several times during the seasonal peak in malaria transmission (IPTc) has been investigated as a method of preventing malaria.

A Cochrane review published in 2008 reviewed the efficacy and safety of chemoprophylaxis and IPT in children [6]. However, although there is some overlap in the mechanism of protection provided by chemoprophylaxis and IPT, these two approaches set out to produce different blood concentration profiles and the efficacy and safety of the two methods may differ. Since the Cochrane review was undertaken, additional IPTc studies have been conducted in different settings using several different drug regimens. A systematic review and meta-analysis was therefore carried out to review new and existing data on the safety and efficacy of IPTc administered seasonally to children under five years of age. We specifically reviewed randomised controlled trials that assessed the efficacy of IPTc against clinical malaria, severe malaria, all-cause hospital admissions, *Plasmodium falciparum* parasitaemia and anaemia when administered to children under five years of age compared to placebo or no intervention. The same studies were also used to assess the effect of IPTc on the prevalence of markers of resistance to SP, as well as any rebound effect in clinical malaria or anaemia in the year following IPTc administration. Non-controlled studies were used additionally to assess the efficacy of IPTc against all-cause mortality and to assess the toxicity of the drugs used.

## Methods

### Search Strategy and Selection Criteria

A systematic literature search for IPTc studies (published, unpublished and ongoing) was carried out on 25th March 2009 and updated on 19th August 2010. A single investigator (ALW) developed and conducted the search. Studies were identified using database searches, citation searches of selected articles and contact with investigators. The electronic databases searched were: Pubmed (1965-present), Web of Science (1970-present), and Global Health (1910-present). Articles in English, German or French were selected for review and no date restrictions were applied to the search. The search was conducted using free-text terms and standardised subject terms appropriate to the specific database. Combinations of the following search terms were used; intermittent, prevention, presumptive, season, therapy/treatment, malaria and mass drug administration (Table S1). Ongoing and completed clinical trials were identified through searches of clinical trial databases [7], [8].

Study eligibility was assessed by a single investigator (ALW) in an un-blinded manner. Studies were retained if they met the following criteria; i) seasonal administration of more than one therapeutic course of anti-malarial drugs, whether or not subjects were known to be infected, ii) study subjects were children, aged less than 5 years, resident in a malaria endemic area, and, iii) the objective of the study was evaluation of the effect of drug administration on clinical malaria (with parasitological confirmation). Studies which enrolled children older than 5 years were included if data on children under 5 years were available for analysis. All study designs were accepted, although only controlled studies were used for analysis of primary efficacy outcomes. Studies were excluded if their aim was to evaluate; i) the effect of a sustained protective drug concentration against infection (chemoprophylaxis), or, ii) the effect of drug administration on interruption of transmission (mass drug administration). Studies in population subgroups such as anaemic children were also excluded.

### Data Extraction and Outcome Measures

Data from eligible studies was extracted based on the intention-to-treat principle into a purpose built database by a single investigator (ALW) (Excel, Microsoft, 2007). We did not have access to data from individual children and, instead, combined results taken from published papers were used. Investigators were contacted directly if any information was unclear or not specified in the published articles.

Clinical malaria was defined as an illness accompanied by an axillary temperature of greater than or equal to 37.5°C or a history of fever within the previous 24 or 48 hours and the presence of asexual forms of *P. falciparum* parasitaemia at any density. An alternative definition of clinical malaria using a locally defined threshold concentration of *P. falciparum* parasitaemia was also used for comparison. Severe malaria was defined as per the WHO definition [9]. Anaemia was defined as a haemoglobin (Hb) concentration less than 8g/dL or an haematocrit (Hct) of less than 25%. Parasitaemia was defined as the presence of asexual forms of *P. falciparum* at any density. Molecular markers of SP resistance assessed were the *dhfr* triple (51, 59, 108) mutations and the *dhps* 437 mutation, associated with resistance to pyrimethamine and sulphones respectively. Adverse events (AE) and Serious Adverse Events (SAE) were defined as per standard ICH definitions [10].

All trials used in the meta-analysis, whether published or unpublished, had been approved by an ethics committee. The risk of bias in the studies was assessed by a single investigator (ALW) in an un-blinded manner using a tool developed by the Cochrane Collaboration (focusing on specific domains including sequence generation, allocation concealment, blinding, incomplete outcome data and selective outcome reporting) [11]. Due to the small number of studies identified, trials were not excluded based on quality assessment and sensitivity analysis was not performed. For the same reason, we were not able to formally assess for publication bias.

### Data Analysis

The effect of IPTc on malaria incidence (total number of episodes) was expressed as a rate ratio. Person time at risk was expressed in years (Person Years at Risk, PYAR) and was defined as date of exit from the study minus date of enrolment into the study. Date of exit for children who died was the date of death and was defined as the date of the last contact with active or passive malaria surveillance systems if a child was lost to follow up. Person time at risk was reduced by 28 days (21 days in two studies [12], [13]) for each episode of clinical malaria treated according to national guidelines with an effective anti-malarial drug, thus reducing the likelihood of recrudescence affecting the rate ratio. Only crude rates were used, as opposed to those adjusted for covariates, such as usage of insecticide treated bednets (ITN) or age. Confidence intervals (CI) were calculated using an approximate standard error obtained from the summary data on the total number of clinical malaria episodes and PYAR. 95% CI and P-values were calculated using standard formulae [14]. Protective efficacy was calculated as PE = 1−(Rate ratio of clinical malaria during the intervention period)×100%. The difference between rates in the IPTc and control arms was calculated. Due to the heterogeneity of the efficacy estimates from the studies, a summary effect measure was calculated using random effect meta-analysis with inverse-variance weights. Forest plots also depict a summary effect measure calculated using fixed effect meta-analysis for comparison. Fixed effect meta-analysis considers that variation between studies is due purely to random variation. Random effect meta-analysis assumes a different underlying effect for each study and takes this into account as an additional source of variation, resulting in a summary effect measure with wider CI. Where studies are heterogeneous it is more appropriate to use a random effect model [15]. Heterogeneity between trials was quantified using the I^2^ statistic [16]. The effect of IPTc on the prevalence of anaemia and of parasitaemia, as well as the mean Hb or Hct concentration was assessed and 95% CI and two-sided P-values were calculated using standard methods [14]. Deaths occurring during the intervention period were expressed per 1000 children who received the first dose of at least one course of IPTc and per 1000 PYAR, with the 95% CI and P-value for the corresponding relative risk and rate ratio calculated using standard methods [14]. The most common AEs were pooled across studies for each IPTc regimen and expressed per 1000 courses of IPTc delivered. Incidence of AEs was then compared to that in the control arms using a z-test. Statistical analysis was conducted using Excel (Microsoft, 2007) and STATA 10 (StataCorp, Texas, U.S.A.). This review has been reported according to PRISMA guidelines [17], [18] (Table S2).

## Results

### Characteristics of Studies Identified

**Figure 1:**
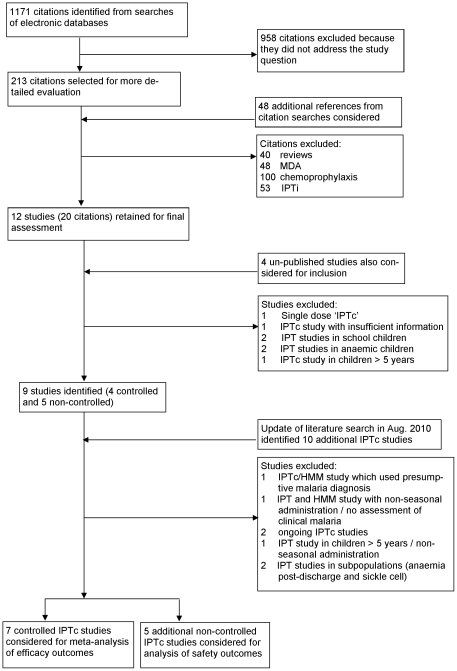
Flow Diagram of Study Selection Process.

The systematic literature search identified seven controlled IPTc studies [12], [13], [19], [20], [21], [22], [23] and a further five studies which were not controlled [24], [25], [26], [27], [28] (Figure 1). Characteristics of controlled and non-controlled studies are shown in Table 1 and Table 2, respectively. Studies which were excluded from the review are described in Table S3.

**Table 1:** Characteristics of Controlled Studies.

	Cisse (2006) [20]	Kweku (2008) [22]	Dicko (2008) [21]	Bojang (2010) [19]	Dicko (2011) [12]	Konaté (2011) [13]	Sesay (2011) [23]
Location	Niakhar, Senegal	Hohoe, Ghana	Kambila, Mali	Basse, Gambia	Kati Region, Mali	Boussé, Burkina Faso	Farafenni Gambia
Entomological inoculation rate (per person per year)	10	65	137	1–50	7–37	11–74	<10
Bednet usage	22% any net	11% ITN	<5% any net	68% ITN	>99% ITN	93% ITN	93% ITN
IPTc Drugs	AS+SP	SP bimonthly AS+AQ bimonthly, AS+AQ	SP bimonthly	SP+AQ, SP+PQ, DHA+PQ	SP+AQ	SP+AQ	SP+AQ
Delivery	Trial staff at facility	CHW	Trial staff at facility	Trial staff at facility	Trial staff at facility	Trial staff at facility	CHW
Control	Placebo	Placebo	Nothing	Non-randomised arm	Placebo & ITN	Placebo & home management of malaria	

AQ: amodiaquine, AS: artesunate, CHW: community health worker, DHA: dihydroartemisinin, ITN: insecticide treated bednet, PQ: piperaquine, SP: sulphadoxine pyrimethamine.

**Table 2:** Characteristics of Non-controlled Studies.

	Sokhna (2008) [24]	Cisse (2009) [25]	Bojang (2011) [26]	Kweku (2009) [27]	Cisse (unpublished) [28]
Location	Niakhar, Senegal	Ndoffane, Senegal	Basse, The Gambia	Jasikan, Ghana	Tivaouane, Senegal
Entomological inoculation rate (per person per year)	10	Not known	1–50	65	10
Bednet usage	18% slept under intact or impregnated net	29% slept under intact or impregnated net	50%/62% slept under intact or impregnated net in Reproductive and Child Health trekking team and CHW arms, respectively	19% bednet usage, 14% ITN usage	28% bednet usage, 13% ITN usage
IPTc Drugs	SP+1AS monthlySP+3AS monthlyAQ+3AS monthlySP+AQ monthly	SP+AQ monthlyDHA+PQ monthlySP+PQ monthly	SP+AQ monthly	SP+AQ (May/Jun./Sept./Oct)	SP+AQ monthly
Delivery	Study staff	CHW	CHW or Reproductive and Child Health trekking team	Health facility (routine staff with support) or CHW	CHW

AQ: amodiaquine, AS: artesunate, CHW: community health worker, DHA: dihydroartemisinin, ITN: insecticide treated bednet, PQ: piperaquine, SP: sulphadoxine pyrimethamine.

All seven controlled trials were conducted in West Africa in areas of seasonal malaria transmission, although the transmission in the two sites in Ghana was perennial with seasonal peaks (Table 1). The estimated Entomological Inoculation Rate in the study areas ranged from less than 10 up to 137 infectious bites per person per year. The incidence of malaria in the placebo group during the transmission season was relatively low in Farafenni and Basse, The Gambia and Hohoe, Ghana but higher in Kati Region and Kambila, Mali, Boussé, Burkina Faso and Niakhar, Senegal. Usage of bednets was low (<5–22%) in three study sites [20], [21], [22] and, at these sites, nets were usually in a bad condition or un-treated. However, in Basse [19] and Farafenni [23] usage of ITNs was relatively high (68% and 93%, respectively). In two studies conducted in Boussé [13] and Kati Region [12], all study participants received ITNs on entry into the study and surveys found ITN usage to be over 93% and 99%, respectively.

IPTc was administered either monthly or every 2 months and the number of courses (between 2 and 6) was tailored to the duration of the malaria transmission season. In all studies, except for one (Tivaouane), the intervention was implemented for only one transmission season. A variety of anti-malarial drugs were used, either alone or in combination. Standard adult tablets were used throughout. All studies reported that study drugs had greater than 90% efficacy when used for treatment of patients (children or adults) with uncomplicated but symptomatic malaria in the study area. Two efficacy studies (Hohoe [22] and Farafenni [23]) utilised community health workers (CHWs) to deliver IPTc; in the other studies, IPTc was delivered in routine health facilities by dedicated study staff. In all efficacy studies, over 75% of children received at least the first dose of each IPTc round.

In the majority of the studies, two cross-sectional surveys were carried out during the intervention year; one at baseline and another at the end of the intervention period. Either passive [19], [22], [23] or both passive and active case detection [12], [13], [20], [21] took place during the intervention period, which began at the time of administration of the first IPTc course and ended 4–6 weeks after the last IPTc course had been given. Surveillance was continued through the intervening dry season in four studies (Boussé, Hohoe, Kambila and Kati [12], [13], [21], [22]) and during the subsequent malaria transmission season in five (Boussé, Hohoe, Kambila, Kati and Niakhar [12], [13], [20], [21], [22]), although data for the second year of follow-up for the Boussé and Kati Region trials were not yet available at the time of data analysis. Methods used to assess the safety of IPTc varied across studies. In all studies, physicians were available to assess any events which arose during the period of follow up. In addition, all children, or a random sample of children, were questioned or a standardised questionnaire was administered following the first or all courses of IPTc to identify any AEs. In one study (Niakhar), children were physically examined. In the Kambila study, only the incidence of SAEs was recorded.

Controlled studies were generally of high quality (Table 3). Four studies were placebo controlled and double blind (Niakhar [20]; Boussé [13]; Kati Region [12] and Farafenni [23]). In one placebo-controlled study (Hohoe) [22] the lead investigator was accidentally un-blinded. In another study (Kambila [21]) children in the control arm received no intervention. In both these cases there may have been potential for introduction of bias. In Basse [19], a non-randomised cohort of children of the same age group from neighbouring villages was used as a reference group for comparisons of malaria incidence and safety. In this study, there was also potential for introduction of bias due to a higher loss to follow up in the SP+amodiaquine (AQ) arm (22%) compared to the other arms (11–12%). The reason for this difference in attrition is unclear since there was no obvious difference in the characteristics of the groups of children and the incidence of AEs in children in the SP+AQ arm was similar to that recorded in children in the other arms of the trial. Non-controlled trials were all non-blinded and so there was a risk of introduction of bias in these studies.

**Table 3:** Assessment of risk of bias in studies.

	Study Site	Adequate sequence generation	Allocation assignment	Blinding of participants, personnel and outcome assessors	No evidence of incomplete outcome data	No evidence of selective outcome reporting
Cisse (2006) [20]	Niakhar	Yes	Yes	Yes (double blind)	Yes	Yes
Kweku (2008) [22]	Hohoe	Yes	Yes	No (drug packers and principal investigator un-blinded)	Yes	Yes
Dicko (2008) [21]	Kambila	Yes	Yes	No (open label, control arm received no intervention)	Yes	Yes
Bojang (2010) [19]	Basse	No (control arm for malaria incidence/safety comparison non randomised)	Yes	No (open label)	No (higher loss to follow up in SP+AQ arm, 22% vs 11–12%, no obvious explanation)	Yes
Dicko (2011) [12]	Kati Region	Yes	Yes	Yes (double blind)	Yes	Yes
Konaté (2011) [13]	Boussé	Yes	Yes	Yes (double blind)	Yes	Yes
Sesay (2011) [23]	Farafenni	Not assessed (information not available)				
Sokhna (2008) [24]	Niakhar	No (villages chosen for logistical convenience, systematic allocation of individual children)		No (open label)	Yes	Yes
Cisse (2009) [25]	Ndoffane	Yes	Yes	No (open label)	Yes	Yes
Bojang (2011) [26]	Basse	Yes	Yes	No (open label)	Yes	Yes
Kweku (2009) [27]	Jasikan	Yes	Yes	No (open label)	Yes	Yes
Cisse [28] (unpublished)	Tivaouane	Not assessed (information not available)				

AQ: amodiaquine, SP: sulphadoxine pyrimethamine.

### Efficacy of IPTc

The PE of IPTc with SP monotherapy administered every 2 months against uncomplicated clinical malaria (any parasite density) was 35% (95%CI: 17–48%) in Ghana and 69% (95%CI: 54–80%) in Mali (Table 4). Artesunate (AS)+AQ administered bimonthly in Ghana had the lowest PE of any drug regimen investigated (31%; 95%CI: 13–46%), but this drug regimen administered monthly had a much higher PE (75%; 95%CI: 65–82%). SP+AS had a PE of 83% (95%CI: 79–86%) in Senegal. The PE of SP+AQ given monthly was consistently high, ranging from 71% (95%CI: 68–74%) in Burkina Faso, to 93% (95%CI: 79–97%) in The Gambia. The PE of SP+piperaquine (PQ) in The Gambia was 93% (95%CI: 80–97%). The PE of SP+AQ administered monthly in the Kati and Boussé region trials, in which ITNs were also given to each child at the onset of the trial, was 83% (95%CI: 80–86%) and 71% (95%CI: 68–74%), respectively. The very low number of malaria episodes in Farafenni precluded any meaningful interpretation of the effect of IPTc (SP+AQ) on clinical malaria in this study (PE: 49%, 95%CI: −459–95%). The rate difference was highest in Kambila, Mali at 4544 episodes per 1000 children due to the high incidence of clinical malaria in the control arm in this study. The rate difference was also high in Niakhar, Boussé and Kati Region at 3681, 2671 and 1996 clinical malaria episodes per 1000 PYAR, respectively. The rate difference was lowest in Farafenni at 6 episodes per 1000 PYAR.

**Table 4:** Effect of IPTc on clinical malaria during the intervention period.

	Study Site	Drug Regimen	No. of children	Definition of clinical malaria (fever or history of fever plus any level of parasitaemia)			
				No. of episodes^A^	Incidence Rate per 1000 PYAR	Rate Difference per 1000 PYAR	PE (95% CI)
Cisse (2006) [20]	Niakhar	SP+AS	542	96	758	3681	83 (79–86)
		Placebo	546	438	4439		-
Kweku (2008) [22]	Hohoe	SP bimonthly	613	112	403	214	35 (17–48)
		AS+AQ bimonthly	562	109	425	192	31 (13–46)
		AS+AQ monthly	626	44	153	464	75 (65–82)
		Placebo	650	183	617		-
Dicko (2008) [21]	Kambila	SP bimonthly	58	30	2000	4544	69 (54–80)
		Control	59	86	6544		-
Bojang (2010) [19]	Basse	SP+AQ	336	4	58	733	93 (79–97)
		SP+PQ	336	4	57	734	93 (80–97)
		DHA+PQ	336	7	103	688	87 (71–94)
		Control	286	41	791		-
Dicko (2011) [12]	Kati Region	SP+AQ	1509	149	411	1996	83 (80–86)
		Placebo	1508	832	2407		-
Konaté (2011) [13]	Boussé	SP+AQ	1509	416	1114	2671	71 (67–74)
		Placebo	1505	1232	3785		-
Sesay (2011) [23]	Farafenni	SP+AQ	639	1	5	6	49 (−459–95)
		Placebo	638	2	11		

A Total number of clinical malaria episodes, AQ: amodiaquine, AS: artesunate, CI: confidence interval, DHA: dihydroartemisinin, PE: protective efficacy, PQ: piperaquine, PYAR: person years at risk, SP: sulphadoxine pyrimethamine.

**Figure 2:**
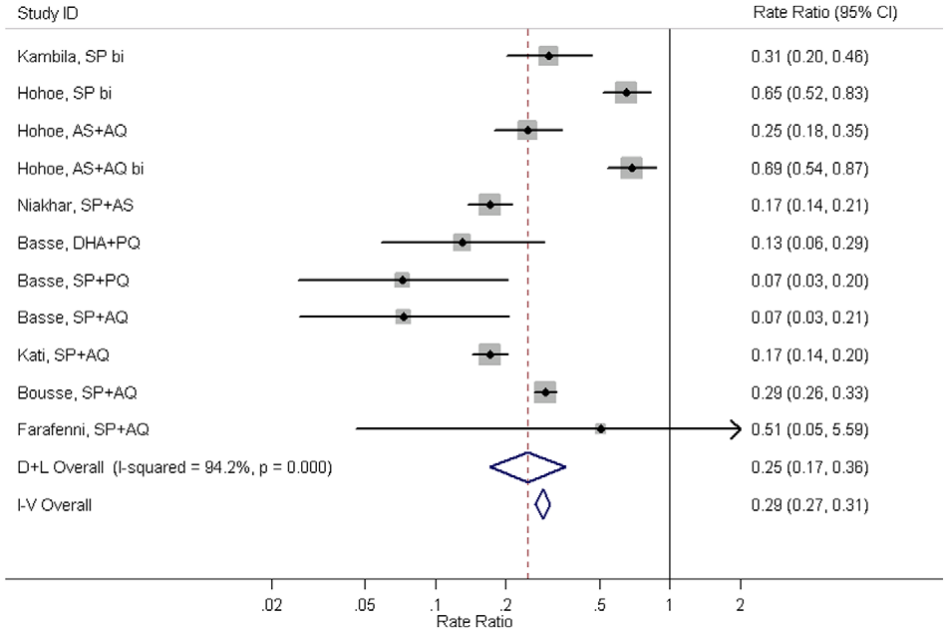
Effect of IPTc on clinical malaria (any level of parasitaemia) during the intervention period. NOTE: D+L Overall = Random effect meta-analysis, I–V Overall = Fixed effect meta-analysis. AQ: amodiaquine, AS: artesunate, bi: bimonthly administration, CI: confidence interval, DHA: dihydroartemisinin, PQ: piperaquine, SP: sulphadoxine pyrimethamine.

Random effect meta-analysis of all studies gave a summary rate ratio of 0.25 (95%CI: 0.17–0.36) (Figure 2) and restricting the meta-analysis to studies in which IPTc was delivered monthly gave a summary rate ratio of 0.18 (95%CI: 0.13–0.25, p<0.001) which is equivalent to a summary PE of 82% (95%CI: 75–87%). Use of fixed effect meta-analysis or a more specific malaria case definition which included a locally defined parasite threshold did not alter the results appreciably (data not shown).

Data on severe malaria were available only for two trials (Kati Region [12] and Boussé [13]) which were conducted in parallel and designed to have sufficient power to assess this outcome when the data were pooled. There were 4 and 22 cases of severe malaria detected in intervention and placebo groups, respectively, giving a PE of 82% (95%CI: 48–94%, p = 0.002). There was also evidence of a substantial reduction in the incidence of all-cause hospital admissions in these two trials; 27 children were admitted to hospital in the IPTc group compared with 45 in the placebo group (PE: 41%, 95%CI: 5–63%, p = 0.03).

Pooling of data across twelve studies [12], [13], [19], [20], [21], [22], [23], [24], [25], [26], [27], [28] indicated that there were 47 deaths (all-cause) among 35,350 children in the IPTc arms compared to 16 deaths per 5,186 children in the control arms during the intervention period. This gives a PE of IPTc against all-cause mortality of 57% (95%CI: 24–76%, p = 0.003) during the intervention period. Pooling of mortality data across eight studies [12], [13], [19], [20], [21], [22], [26], [27] which reported person time at risk, showed that children receiving IPTc had 0.51 times the rate of all-cause mortality compared to children in control arms during the intervention period (95%CI 0.28–0.95, p = 0.03).

In the trials conducted in Kati Region and Boussé [12], [13], children who received IPTc with SP+AQ had a significantly lower prevalence of anaemia and higher mean Hb concentration at the end of the intervention period compared to children who received placebo (Table 5). In contrast to these findings, no significant difference in mean Hb/Hct concentration was observed at the end of the intervention period in the three other studies which assessed this endpoint [21], [22], [23]. Meta-analysis of the prevalence of anaemia in five studies showed no overall positive effect of IPTc (Risk ratio: 0.84, 95%CI: 0.59–1.21, p = 0.36) [12], [13], [20], [21], [22].

**Table 5:** Effect of IPTc on mean haemoglobin/haematocrit concentrations in intervention and control arms at the end of the intervention period.

	Study Site	Drug Regimen	No. children	Mean Hb (g/dL)/Hct (%) concentration [95% CI]	p-value (z-test)
Kweku (2008) [22]	Hohoe	SP bimonthly	550	9.2 (6.6–11.8)	0.20
		AS+AQ bimonthly	464	9.2 (6.6–11.8)	0.23
		AS+AQ monthly	559	9.4 (7.0–11.8)	0.18
		Placebo	589	9.3 (6.7–11.9)	-
Dicko (2008) [21]	Kambila	SP bimonthly	55	32.1 (31.2–33.0)	0.80
		Control	54	31.9 (31.0–32.8)	-
Dicko (2011) [12]	Kati Region	SP+AQ	1422	11.0 (10.7–11.2)	0.06
		Placebo	1433	10.7 (10.5–10.9)	-
Konaté (2011) [13]	Boussé	SP+AQ	1444	11.0 (10.9–11.1)	<0.001
		Placebo	1441	10.4 (10.3–10.4)	-
Sesay (2011) [23]	Farafenni	SP+AQ	513	10.2 (7.1–13.3)	0.79
		Placebo	533	10.3 (7.4–13.2)	-

AQ: amodiaquine, AS: artesunate, CI: confidence interval, DHA: dihydroartemisinin, Hb: haemoglobin, Hct: haematocrit, PQ: piperaquine, SP: sulphadoxine pyrimethamine.

In three studies [12], [13], [20], there was a highly significant reduction in the prevalence of parasitaemia in the IPTc arm at the end of the intervention period (Table 6). In an additional study [21], there was evidence of a lower prevalence of parasitaemia in the IPTc arm compared to the control but the sample sizes were relatively small. Data from Hohoe [22] indicated that bimonthly administered IPTc was less effective at reducing parasitaemia at the end of the intervention period compared to monthly administered IPTc. In one study conducted in Farafenni, The Gambia [23] the prevalence of parasitaemia was very low and was similar among IPTc and control children at both the pre- and post-intervention surveys. Meta-analysis of the prevalence of parasitaemia in six studies which assessed this endpoint showed a positive effect of IPTc (Risk ratio: 0.47, 95%CI: 0.30–0.75, p = 0.002) [12], [13], [20], [21], [22], [23].

**Table 6:** Effect of IPTc on prevalence of parasitaemia, *dhps* and *dhfr* resistance markers measured at cross sectional surveys during the intervention year.

	Study	Arm	Children with asexual Parasitaemia^A^ % (N)	p-value^B^	Parasite carriage of resistance markers				Estimated minimum prevalence of resistant parasitaemia amongst children	
					*dhfr* triple mutation % (N)	p-value^B^	*dhps* 437 mutation % (N)	p-value^B^	*dhfr triple* mutation %	*dhps* 437 mutation %
Pre-intervention										
Cisse (2006) [20]	Niakhar	SP+AS	37 (516)	0.77	51 (71)	0.06	28 (67)	0.92	19	10
		Placebo	36 (512)	-	67 (69)	-	29 (72)	-	24	11
Dicko (2008) [21]	Kambila	SP bimonthly	36 (53)	0.14	-	-	-	-	-	-
		Control	23 (53)	-	-	-	-	-	-	-
Dicko (2011) [12]	Kati Region	SP+AQ	-	-	59 (41)	-	38 (41)	-	-	-
		Placebo	-	-					-	-
Konaté (2011) [13]	Boussé	SP+AQ	-	-	33 (132)	-		-	-	-
		Placebo	-	-					-	-
Sesay (2011) [23]	Farafenni	SP+AQ	0.5 (639)	0.99	-	-	-	-	-	-
		Placebo	0.5 (638)	-	-	-	-	-	-	-
Post-intervention										
Cisse (2006) [20]	Niakhar	SP+AS	14 (440)	<0.001	95 (41)	0.01	86 (28)	<0.001	13	12
		Placebo	37 (446)	-	75 (122)	-	44 (89)	-	28	16
Kweku (2008) [22]	Hohoe	SP bimonthly	16 (550)	0.14	90 (51)	0.41	63 (54)	0.33	14	10
		AS+AQ bimonthly	22 (464)	0.29	84 (55)	0.86	63 (51)	0.35	18	14
		AS+AQ monthly	5 (559)	<0.001	92 (13)	0.49	45 (11)	0.62	4	2
		Placebo	20 (589)	-	85 (53)	-	54 (54)	-	17	11
Dicko (2008) [21]	Kambila	SP bimonthly	4 (55)	0.04	-	-	-	-	-	-
		Control	15 (54)	-	-	-	-	-	-	-
Dicko (2011) [12]	Kati Region	SP+AQ	7 (1405)	<0.001	49 (83)	0.02	68 (83)	<0.001	5	6
		Placebo	13 (1423)	-	34 (160)		44 (165)		6	8
Konaté (2011) [13]	Boussé	SP+AQ	11 (1436)	<0.001	50 (114)	0.61			6	-
		Placebo	42 (1430)	-	53 (122)				22	-
Sesay (2011) [23]	Farafenni	SP+AQ	0.6 (513)	0.52	-	-	-	-	-	-
		Placebo	0.9 (533)	-	-	-	-	-	-	-

A Any density parasitaemia,

B Chi^2^ test, AQ: amodiaquine, AS: artesunate, DHA: dihydroartemisinin, N = total number of samples analysed, PQ: piperaquine, SP: sulphadoxine pyrimethamine.

### Safety of IPTc

No drug-related SAEs were observed during the intervention period in which a total of 100,767 IPTc courses were delivered to 35,350 children. No cases of Stevens Johnson syndrome were seen in the 32,757 children who received SP or SP-containing regimens, and no cases of serious blood dyscrasias or liver damage in the 31,327 children who received AQ-containing combinations.

The prevalence of AEs varied greatly between individual studies, especially for vomiting which was the most commonly reported AE. Thus, care is needed in the interpretation of the results of comparisons between treatment regimens when combined data from several trials are used. Nevertheless, some general observations can be made (Table S4). Vomiting was reported most frequently following administration of SP+PQ, DHA+PQ and SP+AQ. Fever, headache and pruritus were also reported more frequently following administration of these regimens. Administration of SP+PQ and DHA+PQ was associated with increased reports of minor skin rash and coughing compared to children in control arms. AS+AQ was associated with a low incidence of AEs, with only reports of drowsiness and abdominal pain being significantly raised compared to control children. SP+AS was well tolerated and was associated with the lowest incidence of AEs overall.

In studies in which alternative drug regimens were compared, vomiting was reported more frequently following administration of AQ- containing regimens than other regimens. For example in Ndoffane, Senegal, vomiting was reported more frequently following administration of SP+AQ than DHA+PQ or SP+PQ [25].

A concern of any form of community wide drug administration is that it will encourage the emergence of drug resistant parasites. In the study conducted in Niakhar, the prevalence of the *dhfr* triple mutation and the *dhps* mutation increased substantially in both IPTc and placebo arms during the intervention period (Table 6) [20]. At the post-intervention survey, the proportion of parasitaemic children carrying parasites with the *dhfr* triple mutation and the *dhps* mutation was higher in children in the SP+AS arm than in the placebo arm (*dhfr*: SP+AS 95%, placebo 75%, p = 0.01 and *dhps*: SP+AS 86%, placebo 44%, p<0.001). However, as the prevalence of parasitaemia in the children who received IPTc was overall much lower than that in children in the placebo group, the estimated prevalence of drug resistant parasitaemia among study children was lower in the SP+AS arm than in the placebo arm (*dhfr*: SP+AS 13%, placebo 28% and *dhps*: SP+AS 12%, placebo 16%). Data on the prevalence of drug resistance markers in the second year of follow-up was only available for the study conducted in Niakhar. In this study, the difference in the prevalence of markers of resistance to SP was lost at the end of the second year of follow-up (*dhfr*: SP+AS 88%, placebo 86%, p = 0.69 and *dhps*: SP+AS 64%, placebo 77%, p = 0.10). In the Kati Region, the prevalence of the *dhfr* triple and *dhps* mutations was higher in the IPTc arm compared to the placebo arm at the post intervention survey [12]. However, the estimated prevalence of drug resistant parasitaemia among study children was comparable in the SP+AQ arm and placebo arms (*dhfr* triple mutation SP+AQ 5%, placebo 6% and *dhps* 437 mutation SP+AS 6%, placebo 8%). In a further two studies (Boussé and Hohoe), the proportion of parasites carrying SP resistance markers was similar in IPTc and placebo arms at the post-intervention survey [13], [22].

**Figure 3:**
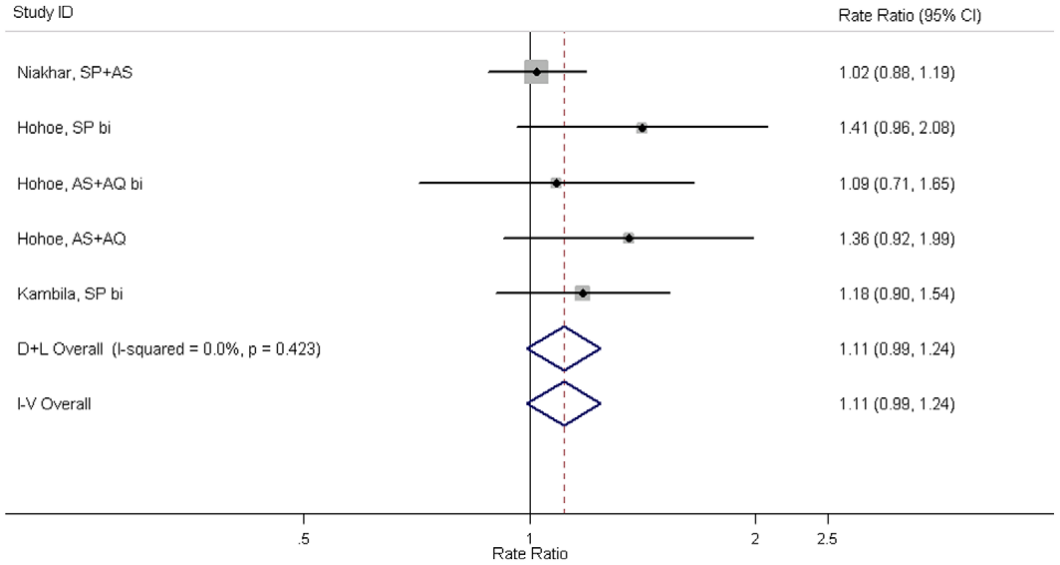
Effect of IPTc on clinical malaria (any level of parasitaemia) during the subsequent transmission season (following IPTc administration). NOTE: D+L Overall = Random effect meta-analysis, I–V Overall = Fixed effect meta-analysis. AQ: amodiaquine, AS: artesunate, bi: bimonthly administration, CI: confidence interval, SP: sulphadoxine pyrimethamine.

A further safety concern for IPTc is that it will impair the development of natural immunity making children more susceptible to malaria after treatment is stopped. At the time of this review, data on clinical outcomes in the year after administration of IPTc were available for only three studies [20], [21], [22]. None of these studies demonstrated a statistically significant increase in the incidence of clinical malaria during the transmission season following the intervention. Random effect meta-analysis gave a summary effect measure of 1.11 (95% CI 0.99–1.24, p = 0.07) (Figure 3). A similar result was obtained when a more specific malaria case definition was used (data not shown). At the end of the malaria transmission season in year 2, the prevalence of anaemia in children who had received SP+AS in Niakhar was higher than in children who had received placebo in year 1 (SP+AS 10%, placebo 6%, p = 0.02) [20]. However, the mean Hb concentration (g/dL, 95%CI) was similar in SP+AS and placebo arms (SP+AS: 9.4 (9.3–9.6), placebo: 9.6 (9.4–9.6), p = 0.24). The prevalence of anaemia was similar in IPTc and placebo arms of the Hohoe trial at the end of the transmission season in the post-intervention year (SP bimonthly 12%, AS+AQ bimonthly 15%, AS+AQ monthly 10%, placebo 12%) [22]. The prevalence of parasitaemia at the end of the transmission season in the post-intervention year was similar among children who received IPTc or placebo in the previous year in the Niakhar and Hohoe studies (Niakhar: SP+AS 28%, placebo 30%, Hohoe: SP bimonthly 39%, AS+AQ bimonthly 40%, AS+AQ monthly 42%, placebo 40%) [20], [22].

## Discussion

As with any retrospective study of this kind, this review has a number of limitations. It is possible that the literature search may have missed some studies. However, we consider that this is unlikely as the group of scientists involved in the study of IPTc is small and personal enquiries from those known to be active in the field, as well as formal literature searches, were undertaken. Heterogeneity in study design and findings makes some comparisons, for example those on the occurrence of AEs, difficult. Since the reviewers did not have access to individual patient data, the effect of study/patient characteristics, such as baseline parasitaemia, age or ITN usage, on clinical outcomes could not be assessed. Study quality was variable but the small number of IPTc studies identified limited the extent to which sensitivity analysis could be conducted. However, an attempt was made to increase the robustness of the meta-analysis by assessing the effect on PE of using different malaria definitions and using both fixed and random effect meta-analysis. The meta-analysis focused on total malaria episodes, the important public health outcome, rather than first episodes so it may have overestimated PE for this endpoint. Despite these limitations we believe that this review provides a valid overview of current knowledge on the potential of IPTc as a malaria control tool.

All of the controlled studies identified demonstrated a protective effect of IPTc against clinical episodes of malaria during the malaria transmission season ranging from 31% to 93%; the overall PE of IPTc administered monthly was 82%. Analysis of the efficacy trials allows some conclusions to be made about the efficacy of individual drugs and dosing regimens in preventing episodes of clinical malaria. The highest PE was observed using two drugs with a long half-life in combination, SP+AQ or SP+PQ. Long acting drugs used alone or in combination with short acting drugs, such as AS or DHA showed lower PEs. No advantage was seen from the use of artemisinin combination therapies, which should probably be reserved for treatment of clinical malaria when the rapid action of artemisinins is of particular benefit. As would be expected, bimonthly administration of SP or AS+AQ demonstrated a lower PE than monthly IPTc administration. The results add weight to the growing body of evidence which suggests that IPT works largely by providing a period of post-treatment prophylaxis and that the length of this period of protection is determined by the pharmacodynamics of the drugs used [29].

None of the trials was individually powered to demonstrate an effect on severe malaria or all-cause mortality. However, pooled data from Kati Region and Boussé showed a PE of 82% against severe malaria and data from twelve studies suggests that administration of IPTc reduced overall mortality in children aged 3–59 months by more than 50% during the intervention period. These results are supported by a study in The Gambia, which deployed fortnightly chemoprophylaxis with pyrimethamine/dapsone during the malaria transmission season, and demonstrated an approximately 40% reduction in overall mortality in children in the same age range [30]. The PE of IPTc against all-cause mortality over the whole year will have been lower than the PE during the high transmission season, but in countries of the Sahel and sub-Sahel a high proportion of deaths occur during the high transmission season [4], [5], [31], [32]. The studies reviewed here may have underestimated the protective effect of IPTc against all-cause mortality as a result of close monitoring of all study children, including those in control arms. In contrast, effectiveness of IPTc against all-cause mortality may be lower than that observed in controlled studies if IPTc were to be deployed routinely in a public health programme. The apparent impact of IPTc on mortality revealed by this review is impressive. However, these findings should be treated with caution as the number of deaths was small. A large, community randomised trial of IPTc currently under way in Senegal will provide further information on the impact of IPTc on all-cause mortality, although the overall level of malaria transmission has fallen substantially in the study area since the trial was planned and this may reduce the statistical power of this study (B. Cisse, personal communication).

Two of the five studies which assessed the effect of IPTc on the prevalence of anaemia and mean Hb levels demonstrated a beneficial effect. The lack of effect in other studies may have been due in part to the fact that children were kept under close observation, so episodes of malaria or anaemia were detected and treated promptly during the course of the intervention period, and partly due to the varying importance of malaria as a contributory factor to the pathogenesis of anaemia in different epidemiological settings [13].

Three aspects of the safety of IPTc have been considered in this review – immediate toxicity, facilitation of the spread of drug resistance and impairment of naturally acquired immunity.

No SAEs which could be attributed to administration of drugs for IPTc were recorded. In particular, no cases of Stevens Johnson syndrome were observed in the 32,757 children who received SP or SP-containing regimens. These observations confirm reports of the safety of SP when used for IPT in infants [3]. Concerns about the safety of AQ followed the occurrence of serious haematological and hepatic side effects in travellers when the drug was used for chemoprophylaxis. No cases of hepatic failure were recorded in the children included in the studies covered by this review but minor degrees of damage cannot be excluded because liver function tests were not undertaken routinely. A review of a large number of patients included in trials of AQ-containing regimens used for treatment of clinical episodes of malaria in both adults and children concluded that the drug is safe when used in this context [33].

The most commonly recorded AEs were fever and vomiting, which were reported most often following administration of SP+PQ, DHA+PQ and SP+AQ. Vomiting may have been misreported when children spat out AQ or PQ tablets in reaction to their bitter taste. In the study conducted in Jasikan, Ghana [27], there was a reduction in the incidence of AEs at each round of drug administration. In Jasikan, vomiting of the study drugs was successfully mitigated by coaching caretakers to feed the children sugary food at the time of drug administration. It may also be possible to reduce the incidence of vomiting caused by AQ through better dosing and production of tablets with a drug content optimised for use in IPTc. Analysis of data from two IPTc studies conducted in Senegal showed that the incidence of vomiting was most marked in children who were overdosed, because of the dose stratification used, and that it should be possible to reduce the occurrence of vomiting by adjusting the dose [34]. It will be important to minimise the incidence of even minor AEs as these could reduce uptake and compliance with IPTc.

Extensive use of anti-malarials for prevention adds to drug pressure and carries some risk of facilitating the emergence and spread of drug resistant parasites. Drug pressure will be higher when a drug is used for IPTc, covering up to 20% of the population, than when used for IPT in infants or pregnant women (2–5% of the population). Some evidence to support the view that IPTc with SP-containing regimens will select resistant parasites was obtained from the study in Niakhar, Senegal where the proportion of parasites carrying markers of resistance to SP was higher at the end of the malaria transmission season in children who had received IPTc than in children who had received placebo. However, although the proportion was higher, the prevalence of resistant parasitaemia in children who had received IPTc was lower than that in control children because of the effect of IPTc in reducing the overall prevalence of parasitaemia in these children. Which of these variables is most relevant to the spread of drug resistance is uncertain. It was reassuring that by the following year the difference between groups had been lost, suggesting that the parasites carrying resistance markers were at a biological disadvantage in the absence of drug pressure. Nevertheless, if IPTc is sustained for a period of many years it will exert significant drug pressure on the parasite population; use of a combination of drugs rather than a monotherapy should help to mitigate this risk and use of the same drugs for both first-line treatment and IPTc should be avoided whenever possible.

As for any other successful malaria control measure, an effective IPTc regimen will reduce exposure to malaria parasites and thus has the potential to impair the development of naturally acquired immunity. Meta-analysis indicated a small increase in the incidence of clinical malaria episodes during the malaria transmission season in the year following IPTc administration among children who received IPTc compared to control children. In Niakhar, a small increase in the prevalence of anaemia was observed in children who had received IPTc in the previous year but no significant effect on mean Hb concentration was observed so this may have been a chance finding. Unpublished results from Kati Region, Mali and Boussé, Burkina Faso also indicate a slight rebound effect on clinical malaria among children who received IPTc (D. Diallo, personal communication). The 12 month incidence of clinical malaria in the placebo arms of the studies conducted in Kati Region and Boussé indicates that approximately 10% of episodes occur outside the high transmission season. Taking these two factors into account, the PE of IPTc against clinical malaria defined using a parasite threshold of 5000 asexual forms of *P. falciparum* per µL was reduced from 82% during the high transmission season to 63% during the whole 12 month period in Mali and similarly from 70% to 49% during the whole 12 month period in Burkina Faso.

A limitation of the efficacy studies is that IPTc was only administered for only one transmission season. It is likely that if children are given IPTc each year for their first five years of life this will have a greater impact on the development of naturally acquired immunity to malaria. This was observed in The Gambia when fortnightly chemoprophylaxis during the rainy season was given each year for many years [35]. In this Gambian study there was an increase in the incidence of clinical attacks of malaria in the year after the intervention was stopped which was most marked in children who had received chemoprophylaxis during each of the first five years of life. There was, however, no significant rebound in deaths but the number of deaths was small. IPTc may allow greater exposure to malaria parasites than chemoprophylaxis thus facilitating the development of some naturally acquired immunity but if IPTc is widely deployed surveillance for a potential rebound effect will need to be maintained.

Based on the data reviewed, IPTc offers young children a high level of protection against clinical malaria and appears to substantially reduce mortality during the malaria transmission season. Widespread deployment of IPTc will inevitably cause some drug related side effects, may enhance drug resistance and, like all effective malaria control measures, may lead to some loss of naturally acquired immunity. Balancing these benefits and risks will require careful assessment of the burden of malaria in a particular community, its seasonality and the sensitivity of the prevalent parasites to the drugs currently available for use in IPTc. Implementation of IPTc should now be considered as part of an integrated malaria control strategy in areas of seasonal malaria transmission. In considering introduction of IPTc as a malaria control policy it will also be important to assess the acceptability, feasibility, sustainability, cost and cost effectiveness of the intervention. Research that investigates some of these aspects of IPTc is underway [26], [36] and this topic will be the subject of a further review.

## Supporting Information

Table S1 **Search Strategy for Pubmed and Web of Science Databases**
(DOC)

Table S2 **PRISMA Checklist**
(DOC)

Table S3 **IPT Studies Excluded From Review**
(DOC)

Table S4 **Incidence of Most Common Adverse Events During the Intervention Period**
(DOC)
