# Supplementary material for: A Systematic Review and Meta-Analysis of the Efficacy and Safety of Intermittent Preventive Treatment of Malaria in Children (IPTc)
Source: PLoS One. 2011 Feb 14;6(2):e16976. doi: 10.1371/journal.pone.0016976 (PMC3038871; doi:10.1371/journal.pone.0016976)
Supplement: Table S1 — Search Strategy for Pubmed and Web of Science Databases (DOC) [file pone.0016976.s001.doc]

**Table S1**: Search Strategy for Pubmed and Web of Science Databases

| Search Strategy | Search Terms (combined with AND) |
| --- | --- |
| 1 | Intermittent |
|  | prevent* OR PREVENTION & CONTROL |
|  | THERAPY OR treatment |
|  | Malaria OR MALARIA, FALCIPARUM |
| 2 | Season* OR SEASONS |
|  | THERAPY OR treatment |
|  | Malaria OR MALARIA, FALCIPARUM |
|  | (prevent* OR PREVENTION & CONTROL) |
| 3 | Intermittent |
|  | Presumptive |
|  | THERAPY OR treatment |
|  | Malaria OR MALARIA, FALCIPARUM |
| 4 | Mass |
|  | Drug |
|  | Administration |
|  | Malaria OR MALARIA, FALCIPARUM |
