# Supplementary material for: A Systematic Review and Meta-Analysis of the Efficacy and Safety of Intermittent Preventive Treatment of Malaria in Children (IPTc)
Source: PLoS One. 2011 Feb 14;6(2):e16976. doi: 10.1371/journal.pone.0016976 (PMC3038871; doi:10.1371/journal.pone.0016976)
Supplement: Table S3 — IPT Studies Excluded From Review (DOC) [file pone.0016976.s003.doc]

**Table S3**: IPT studies excluded from review

| Study | Title of Study | Reason for exclusion |
| --- | --- | --- |
| Alphonse *et al* (2006) [1] | Single dose sulfadoxine-pyrimethamine or arthemeter lumefantrine in intermittent preventive treatment of malaria in children under five in a high and seasonal malaria transmission area of Burkina Faso | Single dose ‘IPTc’ |
| Clarke *et al* (2008) [2] | Effect of intermittent preventive treatment of malaria on health and education in schoolchildren: a cluster randomised, double-blind, placebo-controlled trial | Non-seasonal administration of IPT  Children enrolled aged > 5 years |
| Nankabirwa *et al* (2010) [3] | Intermittent preventive treatment (IPT) in schoolchildren: a randomised trial to compare the efficacy, safety and tolerability of anti-malarial regimens in Uganda | Non-seasonal administration of IPT  Children enrolled aged > 5 years |
| Verhoef *et al* (2002) [4] | Intermittent administration of iron and sulfadoxine pyrimethamine to control anaemia in Kenyan children: a randomised controlled trial | Specific sub-population (anaemic children) |
| Desai *et al* (2003) [5] | Randomised, controlled trial of daily iron supplementation and intermittent sulfadoxine-pyrimethamine for the treatment of mild childhood anemia in western Kenya | Specific sub-population (anaemic children) |
| Barger *et al* (2009) [6] | Intermittent preventive treatment using artemisinin-based combination therapy reduces malaria morbidity among school-aged children in Mali | Children enrolled aged > 5 years |
| Tagbor *et al* (2010) [7] | Synergistic effects of home-management and intermittent preventive treatment of malaria on malaria morbidity in children aged less than 5 years | Presumptive malaria diagnosis |
| Ahorlu *et al* (2009) [8] | Effectiveness of combined intermittent preventive treatment for children and timely home treatment for malaria control | Non-seasonal administration of IPT  No assessment of clinical malaria |
| Rohner *et al* (2010) [9] | In a randomized controlled trial of iron fortification, anthelmintic treatment, and intermittent preventive treatment of malaria for anemia control in Ivorian children, only anthelmintic treatment shows modest benefit | Non-seasonal administration of IPT  Children enrolled aged > 5 years |
| Bojang *et al* (2010) [10] | Prevention of the recurrence of anaemia in Gambian children following discharge from hospital | Specific sub-population (anaemic children post-discharge) |
| Nakibuuka *et al* (2009) [11] | Presumptive treatment with sulphadoxine-pyrimethamine versus weekly chloroquine for malaria prophylaxis in children with sickle cell anaemia in Uganda: a randomized controlled trial | Specific sub-population (children with sickle cell anaemia) |

________________

1. Alphonse O (2006) Single dose sulfadoxine pyrimethamine or arthemeter lumefantrine in intermittent preventive treatment of malaria in under five children in a high and seasonal malaria transmission area of Burkina Faso. American Journal of Tropical Medicine and Hygiene, 75: p. 1002.

2. Clarke SE, Jukes MC, Njagi JK, Khasakhala L, Cundill B, et al. (2008) Effect of intermittent preventive treatment of malaria on health and education in schoolchildren: a cluster-randomised, double-blind, placebo-controlled trial. Lancet 372: 127-138.

3. Nankabirwa J, Cundill B, Clarke S, Kabatereine N, Rosenthal PJ, et al. (2010) Efficacy, safety, and tolerability of three regimens for prevention of malaria: a randomized, placebo-controlled trial in Ugandan schoolchildren. PLoS One 5: e13438.

4. Verhoef H, West CE, Nzyuko SM, de Vogel S, van der Valk R, et al. (2002) Intermittent administration of iron and sulfadoxine-pyrimethamine to control anaemia in Kenyan children: a randomised controlled trial. Lancet 360: 908-914.

5. Desai MR, Mei JV, Kariuki SK, Wannemuehler KA, Phillips-Howard PA, et al. (2003) Randomized, controlled trial of daily iron supplementation and intermittent sulfadoxine-pyrimethamine for the treatment of mild childhood anemia in western Kenya. J Infect Dis 187: 658-666.

6. Barger B, Maiga H, Traore OB, Tekete M, Tembine I, et al. (2009) Intermittent preventive treatment using artemisinin-based combination therapy reduces malaria morbidity among school-aged children in Mali. Trop Med Int Health 14: 784-791.

7. Tagbor H, Cairns M, Nakwa E, Browne E, Sarkodie B, et al. (2010) The clinical impact of combining intermittent preventive treatment with home management of malaria in children aged below 5 years: cluster randomised trial. Trop Med Int Health [Epub ahead of print].

8. Ahorlu CK, Koram KA, Seakey AK, Weiss MG (2009) Effectiveness of combined intermittent preventive treatment for children and timely home treatment for malaria control. Malar J 8: 292.

9. Rohner F, Zimmermann MB, Amon RJ, Vounatsou P, Tschannen AB, et al. (2010) In a randomized controlled trial of iron fortification, anthelmintic treatment, and intermittent preventive treatment of malaria for anemia control in Ivorian children, only anthelmintic treatment shows modest benefit. J Nutr 140: 635-641.

10. Bojang KA, Milligan PJ, Conway DJ, Sisay-Joof F, Jallow M, et al. (2010) Prevention of the recurrence of anaemia in Gambian children following discharge from hospital. PLoS One 5: e11227.

11. Nakibuuka V, Ndeezi G, Nakiboneka D, Ndugwa CM, Tumwine JK (2009) Presumptive treatment with sulphadoxine-pyrimethamine versus weekly chloroquine for malaria prophylaxis in children with sickle cell anaemia in Uganda: a randomized controlled trial. Malar J 8: 237.
