# Supplementary material for: A Systematic Review and Meta-Analysis of the Efficacy and Safety of Intermittent Preventive Treatment of Malaria in Children (IPTc)
Source: PLoS One. 2011 Feb 14;6(2):e16976. doi: 10.1371/journal.pone.0016976 (PMC3038871; doi:10.1371/journal.pone.0016976)
Supplement: Table S4 — Incidence of Most Common Adverse Events During the Intervention Period (DOC) [file pone.0016976.s004.doc]

**Table S4: Incidence of Most Common Adverse Events (AEs) During the Intervention Period***

| Drug Regimen / Study | | No. of children | Total No of IPTc courses | No of IPTc courses at which AEs were assessed | Adverse Events reported per 1000 courses of IPTc | | | | | | | | | |
| --- | --- | --- | --- | --- | --- | --- | --- | --- | --- | --- | --- | --- | --- | --- |
|  |  |  |  |  | Vomiting | Diarrhoea | Drowsiness | Abdominal pain | Minor skin rash | Headache | | Fever | Pruritus | Cough |
| SP+AQ | Basse | 336 | 930 | 329^A^ | 24 | 55 | 3 | 21 | 3 | 6 | | - | 15 | 70 |
|  | Niakhar | 513 | 1539^C^ | 1527^B^ | 37 | 16 | - | 7 | 3 | 19 | | 37 | - | - |
|  | Ndoffane | 607 | 1693 | 694^A^ | 314 | - | - | - | 20 | 125 | | 316 | 39 | - |
|  | Basse | 12326 | 29810 | 3698^B^ | 37 | 37 | - | 14 | 2 | - | | 68 | 10 |  |
|  | Jasikan | 964 | 3518 | 650^B^ | 118 | 8 | 80 | 2 | - | - | | - | 9 | - |
|  | Kati Region | 1509 | 4342 | 4342 | 4 | 11 | 0.2 | - | 0.5 | - | | 16 | 2 | 13 |
|  | Boussé | 1509 | 3756 | 3756 | 98 | 26 | 0.3 | - | 5 | - | | 48 | 5 | 19 |
|  | **TOTAL** | **17764** | **45588** | **14996** | **59**  p<0.001 | **22**  p<0.001 | **4**  p=0.005 | **5**  p=0.06 | **3**  p=0.94 | **8**  p<0.001 | | **52**  p<0.001 | **7**  p=0.002 | **10**  P=0.1 |
| SP+AS | Niakhar | 535 | 1557 | 450^B^ | 118 | 73 | - | - | 11 | - | | - | 27 | - |
|  | Niakhar | 503 | 1509^C^ | 1512^B^ | 5 | 9 | - | 1 | 1 | 4 | | 17 | - | - |
| SP+3AS | Niakhar | 504 | 1512^C^ | 1503^B^ | 6 | 9 | - | - | 1 | 1 | | 14 | - | - |
|  | **TOTAL** | **1542** | **4578** | **3465** | **20**  p=0.01 | **17**  p=0.68 | **-** | **0.3**  P=0.02 | **2**  p=0.4 | **2**  p=0.004 | | **13**  p<0.001 | **3**  p=0.7 | **-** |
| SP+PQ | Basse | 336 | 964 | 331^A^ | 39 | 33 | 6 | 27 | 24 | 9 | | - | 24 | 106 |
|  | Ndoffane | 569 | 1546 | 454^A^ | 167 | - | - | - | 11 | 40 | | 196 | 18 | - |
|  | **TOTAL** | **905** | **2510** | **785** | **113**  p<0.001 | **14**  p=0.4 | **3**  p=0.8 | **11**  p=0.001 | **17**  p<0.001 | **27**  p<0.001 | | **113**  p<0.001 | **20**  p<0.001 | **45**  p<0.001 |
| DHA+PQ | Basse | 336 | 965 | 325^A^ | 28 | 58 | 3 | 15 | 15 | 3 | | - | 15 | 68 |
|  | Ndoffane | 569 | 1544 | 559^A^ | 136 | - | - | - | 5 | 57 | | 175 | 13 | - |
|  | **TOTAL** | **905** | **2509** | **884** | **96**  p<0.001 | **21**  p=0.5 | **1**  p=0.5 | **6**  p=0.2 | **9**  p=0.008 | **37**  p<0.001 | | **111**  p<0.001 | **14**  p<0.001 | **25**  p=0.002 |
| AS+AQ bi | Hohoe | 562 | 1626 | 1626^B^ | 25 | 27 | 11 | 14 | - | | - | 25 | - | 7 |
| AS+AQ | Hohoe | 626 | 3619 | 3619^B^ | 11 | 10 | 8 | 6 | - | | - | 11 | - | 7 |
| 3AS+AQ | Niakhar | 500 | 1500^C^ | 1485^B^ | 21 | 18 | - | 3 | 1 | | 8 | 33 | - | - |
|  | **TOTAL** | **1688** | **6745** | **6730** | **16**  p=1.0 | **16**  p=0.2 | **7**  P<0.001 | **7**  P<0.001 | **0.1**  p=0.002 | | **2**  p=0.01 | **19**  p=0.03 | **-** | **5**  P<0.001 |
| SP bi | Hohoe | 613 | 1777 | 1777^B^ | **21**  p=0.1 | **21**  p=0.5 | **6**  p=0.004 | **12**  P<0.001 | **-** | | **-** | **26**  p=0.5 | **-** | **15**  p=0.4 |
| Control | Niakhar | 540 | 1581 | 450^B^ | 13 | 80 | - | - | 7 | | - | - | 7 | - |
|  | Hohoe | 650 | 3753 | 3753^B^ | 14 | 11 | 6 | 8 | - | | - | 11 | - | 5 |
|  | Basse | 286 | - | 286^A^ | 49 | 98 | 7 | 35 | 42 | | 21 | - | 38 | 63 |
|  | Kati Region | 1508 | 4304 | 4304 | 2 | 7 | 0 | - | 1 | | - | 15 | 1 | 9 |
|  | Boussé | 1505 | 3512 | 3512 | 34 | 25 | 0.3 | - | 6 | | - | 53 | 9 | 21 |
|  | **TOTAL** | **4489** | **13436** | **12305** | **14** | **16** | **2** | **3** | **3** | | **1** | **21** | **3** | **10** |
| * P-values generated using a z-test to compare the reported incidence of AEs following administration of a particular IPTc drug/combination to the reported incidence in children in control arms, pooled across studies, ^A^AEs assessed in all/sample of children at first IPTc round only, ^B^AEs assessed in all/sample of children at each IPTc round, ^C^Estimate only. Actual number of courses administered not reported, AE: adverse event, AQ: amodiaquine, AS: artesunate, DHA: dihydroartemisinin, PQ: piperaquine, SP: sulphadoxine pyrimethamine | | | | | | | | | | | | | | |
